# Fertility awareness and subclinical infertility among women trying to get pregnant at home

**DOI:** 10.1186/s12905-022-01626-z

**Published:** 2022-02-20

**Authors:** Kaori Iino, Rie Fukuhara, Megumi Yokota, Yoshihito Yokoyama

**Affiliations:** 1 Department of Obstetrics and Gynecology, Hirosaki University Graduate School of Medicine, 5 Zaifu Hirosaki, Hirosaki, Aomori 036-8562 Japan; 2 National Hospital Organization Hirosaki National Hospital, 1 Tominotyo, Hirosaki, Aomori 036-8545 Japan

**Keywords:** Fertility knowledge, Subclinical infertility, Education, Involuntary childlessness, Japanese

## Abstract

**Background:**

Recent studies on fertility awareness among the reproductive population have reported the lack of accurate knowledge about fertility and assisted reproductive technologies. However, there has been little information regarding women trying to get pregnant at home. The aim of this study was to explore the prevalence of subclinical infertility among women trying to get pregnant at home, and to evaluate awareness regarding infertility and reasons for not visiting infertility clinics among women who use pregnancy-assist mobile applications to help them conceive.

**Methods:**

A total of 2084 Japanese women responded to this online survey. We selected 1541 women according to the study criteria. Based on the results of 61 questions, we evaluated knowledge regarding fertility, prevalence of subclinical infertility, and reasons for not visiting the clinic among the participants.

**Results:**

Despite the desire to conceive, the participants had an apparent tendency to overestimate the age limit for childbearing. A total of 338 (21.9%) women answered that in general women aged > 45 years could get pregnant. Approximately 40% of the women had possible subclinical infertility and were unaware of the fact. Additionally, about 70% of the women considered themselves to have infertility problems. Women who were aware of the possibility of infertility hesitated to visit the clinic due to unfamiliarity with a gynecologist or clinic, and apprehensions about the gynecologic examination.

**Conclusions:**

In our study, some women required treatment for infertility. Nonetheless, they hesitated to visit an infertility clinic. Sexual health education, together with proper accessibility to gynecology clinics, are necessary to reduce involuntary childlessness.

## Background

The average age of first pregnancy has increased in most countries since the 1980s [1]. This delay in childbearing has been attributed to changes in women’s social and economic environments, namely, higher education, social progress, and the rise of effective contraception [2–4]. Although the decision of a delayed marriage or pregnancy influences the lifestyle of women, it leads to several health problems. Older mothers have a higher risk of both obstetric and fetal complications, including gestational diabetes, placenta previa, placental abruption, hypertensive disorders of pregnancy, and fetal congenital anomalies [5–8].

Moreover, recent studies on fertility awareness in the reproductive population have reported the lack of accurate knowledge about fertility and assisted reproductive technologies (ART) among women who are trying to conceive [4, 9–22]. The proportion of women having accurate knowledge about fertility is particularly low in Japan, compared to other industrial countries [23]. The importance of health literacy and fertility education has been emphasized in the Japanese society, similar to other countries [21]. Furthermore, the number of websites or applications disseminating information on fertility have rapidly increased in the last decade [20, 24]. In addition, there are a lot of application services that inform the users about the approximate ovulation day from the menstrual cycle, and the number of users of this kind of application is increasing. Although the merit of getting information about reproductive health from websites or smartphone applications could be limited among women who use these daily, the use of self-timing therapy with these application services would be more convenient than going to the clinic.

Thus, women have been able to access fertility information more easily than before. However, the number of infertile patients has been increasing in Japan [25]. The aforementioned facts suggest the presence of other problems, not only the lack of fertility knowledge but also circumstances surrounding women or underlying psychological problems. To clarify the underlying problems, we investigated women who were trying to conceive naturally.

The majority of previous studies on fertility-related knowledge have been conducted among university students [9, 11–14, 17, 22, 26, 27]. These studies concluded that many of university students overestimated women’s age-related decline in ovarian function. Although this tendency of insufficient knowledge of fertility is very important, they could not reveal all the problems. That is because most of university students would not realistically consider fertility and infertility at the time of the survey. Therefore, an investigation among the general population is required.

There have been some studies regarding fertility knowledge among the general population [10, 15, 18, 20, 24, 28, 29]. However, there has been little information on the following aspects among women trying to conceive naturally: (1) prevalence and awareness of subclinical infertility, and (2) reasons for visiting the clinic to identify the possibility of infertility. We performed online research in Japan regarding the knowledge on fertility, the prevalence and risk factors of potential infertility, and reasons for not visiting infertility clinics among women who used pregnancy-assist mobile applications at home.

## Material and methods

### Participants and procedures

We conducted the study on a smartphone application (Lunaluna) that comprised user information, including women’s health, fertility, and ovulation prediction (MTI, Tokyo, Japan) in September 2018 [30]. We chose Lunaluna as the tool for our investigation for the following two reasons. First, Lunaluna is one of the most popular applications presenting women’s health information among Japanese reproductive-aged women. Second, the developers of Lunaluna had experienced other academic research on the application. The time frame that collected the names of participants who wanted to have children was shown for 13 days. At the beginning of the session, the purpose and methods of this study were presented to all participants. Additionally, we informed the participants that this survey was targeted at women trying to get pregnant at that moment. After obtaining informed consent, the participants had to answer several questions on the application site regarding fertility and methods undertaken for childbearing. Each participant took about 50 min to fill in the questionnaire. All participants’ data were anonymized during the analysis. In addition, the data were saved in a computer, which was protected with strict security.

**Fig. 1:**
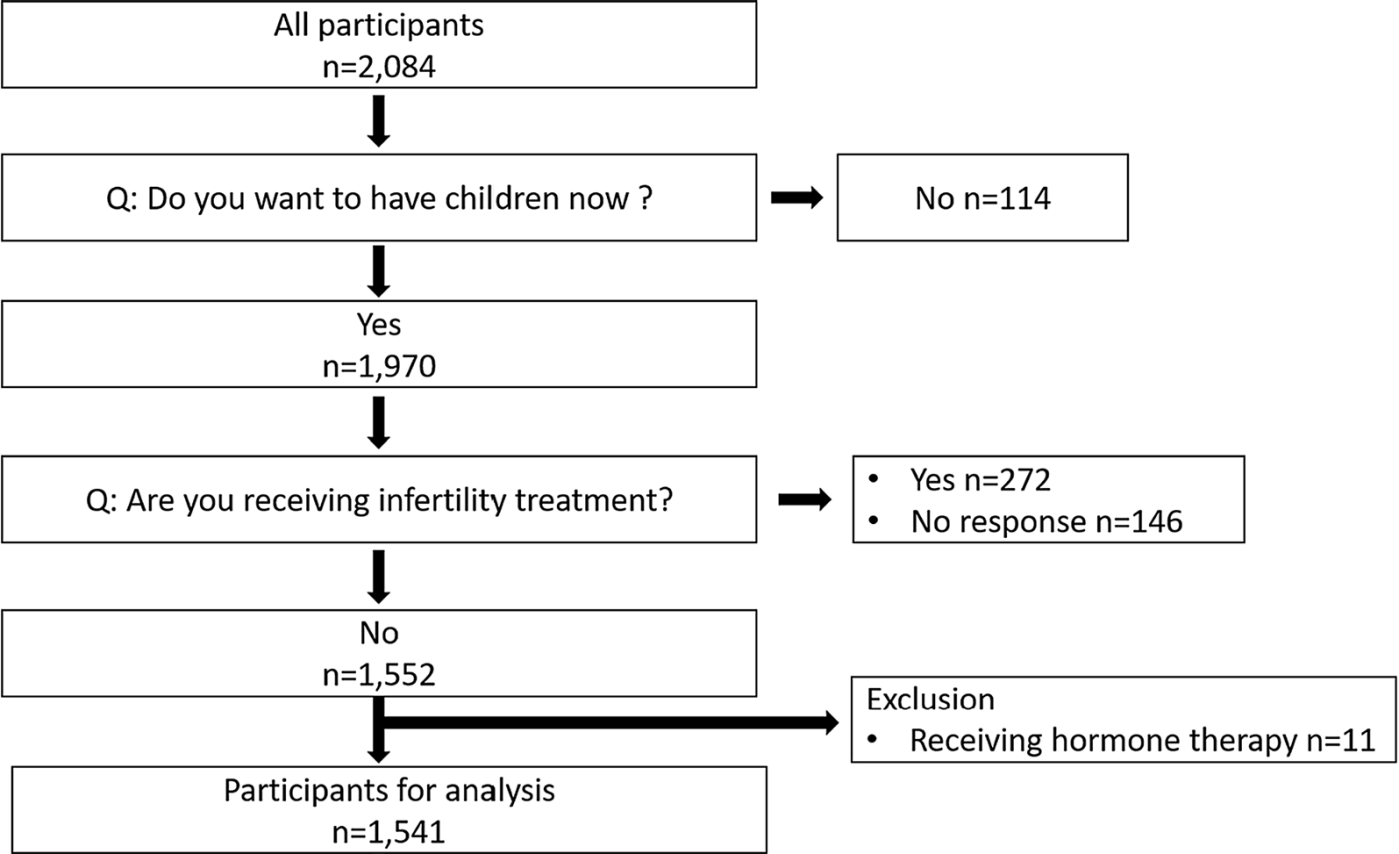
Flow diagram showing participant selection

A total of 2084 women responded to this survey. We selected 1541 women according to the inclusion criterion, that is women who wanted to have children at that time, and two exclusion criteria, that is (1) women receiving infertility treatment and (2) women receiving hormone therapy (Fig. 1). Several questions were asked in order to select the target woman. The first question was as follows: “Do you want to have children now?” We obtained a negative response from 114 women who responded to the remaining questions. Of the 1970 women who wanted to have children, 272 were under treatment for infertility, 146 did not respond to the question on infertility treatment, and 11 were undergoing hormone therapy. We eventually analyzed 1541 participants who completed the questionnaire.

### Measures

Based on previous research, the questionnaire was designed for this study by three gynaecologists, including one reproductive medicine specialists [9, 21, 24]. The questionnaire comprised of 62 questions in seven sections. Every question was shown to the participants in Japanese, and the format of answers to each question was presented in parentheses.

1. Questions for selecting women according to the criteria (3 items)
  The first question was, ‘Do you want to have children now?’ (Yes/No). Only participants who answered ‘Yes’ proceed with the following questions: ‘Are you receiving infertility treatment?’(Yes/No) and ‘Are you receiving hormone therapy?’(Yes/No).
2. Participant’s demographic information (31 items)
  Participants were asked to state their age, body weight, height, smoking status (Never or Former/Current), medical history (open response), social status (Full-time/Part-time/Housewife or Unemployed/Student), marital status (Yes/No), and personal experience of reproduction (Yes/No, if yes, input the number of children).
3. Menstrual information (10 items)
  Participants were asked to state their age of menarche and pattern of menstrual cycle (Regular/Variable-length; persistent ≥ 7-day difference in the length of consecutive cycles/Interval of amenorrhea of ≥ 60 days). The answer to the menstrual cycle was defined according to the criteria of the Stage of Reproductive Aging Workshop [31]. Next, participants were asked whether they had the following symptoms: dysmenorrhea (Yes/No, and if yes, did they use painkillers or not), irregular vaginal bleeding (Yes/No), and hypermenorrhea (Yes/No).
4. Knowledge about age-related decline in fertility (1 item)
  The question was, ‘In general, until what age do you think women can get pregnant?’.
5. Attitude and behavior towards trying to get pregnant (4 items)
  Participants were asked the following questions: ‘Since how long are you having intercourse without contraception?’, ‘How frequent do you have intercourse?’(open response), ‘Do you feel any difficulties while having intercourse?’ (Yes/No), and ‘What are you doing to become pregnant? (Self-timing therapy with application service/Self-timing therapy without application service/Nothing in particular)’. We chose the following question: ‘What are you doing to become pregnant?’ to clarify the number of women who used the application service of self-timing therapy because it would be an important participants’ characteristic for this study.
6. Awareness and attitude regarding infertility (4 items)
  At first, participants were asked the question, ‘Have you ever had an infertility examination?’(Yes/No). Only participants who answered ‘Yes’ proceed with the following questions: ‘What is the likelihood of your infertility?’ (Highly unlikely, Unlikely, Possibly, Likely, and Highly likely) [32], ‘Do you think you should be examined for infertility?’(Yes/No), and ‘Why haven’t you visited a clinic for infertility examination?’(multiple choices allowed: There is no time/I am afraid of the gynecologic examination/I feel afraid of discovering the truth/Partner is not cooperative/I don’t know a familiar gynecologic doctor or clinic/If I am infertile, I don’t want another person to know it/Nothing in particular/ Other, open response).
  With regard to the last question; ‘Why haven’t you visited a clinic for infertility examination?’, we presented several likely answers for the participants to choose from, including the option “other” as it allowed respondents to answer the question freely.
7. Partner’s demographic information (9 items)
  Participants were requested to state their partner’s age, body weight, height, and medical history (open response).

### Statistical analysis

We performed logistic regression analysis to assess the risk factors for subclinical infertility among women trying to get pregnant at home. We defined subclinical infertility among women who failed to conceive successfully after ≥ 12 months of regular, unprotected intercourse (or ≥ 6 months if the women over age 35 years), according to the Practice Committee of the American Society for Reproductive Medicine, 2013 [33]. We calculated the odds ratios (OR) and 95% confidence interval (CI) in the multivariate analysis after simultaneously controlling for potential confounders, such as age, body mass index (BMI), history of smoking, delivery status, incidence of abortion or miscarriage, menstrual cycle, including BMI and smoking history of partner. The multivariate analysis was performed among 1377 women because of 164 missing data. Statistical analyses were performed using the SPSS software package, version 22.0 (SPSS Inc., Chicago, IL, USA). A *P* value < 0.05 was considered statistically significant.

## Results

### Characteristics of participants and partners.

Table 1 summarizes the characteristics of the participants and their partners. The largest age and BMI group of participants were 30–34 years (37.1%) and 18.5–24.9 kg/m^2^ (67.6%), respectively. Regarding their partner, the largest age and BMI group were 30–39 years (52.0%) and 18.5–24.9 kg/m^2^ (64.9%), respectively. 234 (15.2%) participants and 636 their partners (41.3%) were current smokers.

**Table 1:** Characteristics of participants and partners (N = 1541)

Participants’ information		N (%)
Age (years)	16–29	500 (32.4)
	30–34	571 (37.1)
	35–39	314(20.4)
	40–44	128 (8.3)
	45–50	28 (1.8)
Body mass index (kg/m^2^)	< 18.5	225 (14.6)
	18.5–24.9	1040 (67.6)
	25–29.9	198 (12.9)
	≥ 30	76 (4.9)
Smoking	Never smoked or former smoker	1307 (84.8)
	Current smoker	234 (15.2)
Social status	Full-time job	888 (57.6)
	Part-time job	320 (20.8)
	Housewife/Unemployed	323 (21.0)
	Student	10 (0.6)
Marital status	Single	294 (19.1)
	Married	1247 (80.9)
Reproduction	Have children	582 (37.8)
	Experience of abortion or miscarriage	305 (19.8)
History of present illness	Internal Medicine	85 (5.5)
	Gynecologic disease	41 (2.7)
	Endometriosis	12 (0.8)
	Myoma	8 (0.5)
	Ovarian cyst	5 (0.3)
	Dysplasia of uterine cervix	9 (0.6)
	Other	7 (0.5)
	Mental illness	37 (2.4)
	Other	63 (4.1)
	Nothing	1315 (85.3)
Menstrual cycle ^a^	Regular	1185 (76.9)
	Variable length persistent ≥ 7-day difference in length of consecutive cycles	157 (10.2)
	Interval of amenorrhea of ≥ 60 days	72 (4.7)
Dysmenorrhea ^b^	Yes	652 (42.3)
*Partners’ information*		
Age (years)	≤ 29	357 (23.2)
	30–39	801 (52.0)
	40–49	319 (20.7)
	50–64	33 (2.1)
Body mass index (kg/m^2^)	< 18.5	62 (4.0)
	18.5–24.9	1000 (64.9)
	25–29.9	363 (23.6)
	≥ 30	80 (5.2)
Smoking	Never smoked or former smoker	879 (57.0)
	Current smoker	636 (41.3)

^a^Each category was defined according to the criteria of the Stage of Reproductive Aging Workshop

^b^Dysmenorrhea was defined women who need pain killer during menstrual period almost every time

Nearly 80% of the women had a job (full-time: 57.6%; part-time: 20.8%). Of 1541, 1,247 (80.9%) women were married and 582 (37.8%) women had children.

A total of 41 women (2.7%) had gynecologic disease: Endometriosis 12 (0.8%), Myoma 8 (0.5%), Ovarian cyst 5 (0.3%), and Dysplasia of uterine cervix 9 (0.6%). There were no women who had medical history which leads infertility necessarily, for example bilateral oophorectomy or hysterectomy.

Regarding to menstrual cycle, regular, variable-length; persistent ≥ 7-day difference in the length of consecutive cycles, and interval of amenorrhea of ≥ 60 days were 76.9%, 10.2%, 4.7%, respectively. 652 (42.3%) out of all participants suffered with dysmenorrhea.

### Knowledge about the age-related decline of fertility, Attitude and behavior for trying to get pregnant, and Awareness of and attitude towards infertility

Table 2 presents the participants’ knowledge about the age-related decline of fertility, attitude and behavior for trying to get pregnant, and awareness and attitude regarding infertility.

**Table 2:** Questions and answers about Knowledge about the age-related decline of fertility, Attitude and behavior for trying to get pregnant, and Awareness of and attitude towards infertility

Question	Answer	N (%)^a^
In general, what age do you think women can get pregnant until?	< 35 year	55 (3.6)
	35–39 year	412 (26.8)
	40–44 year	735 (47.7)
	≥ 45 year	338 (21.9)
What are you doing to become pregnant?	Self-timing therapy with application service	1146 (74.4)
	Self-timing therapy without application service	50 (3.2)
	Nothing in particular	345 (22.4)
Since how long are you having intercourse without contraception?	< 1 year	746 (48.4)
	1 to < 2 year	267 (17.3)
	2 to < 3 year	159 (10.3)
	3 to < 4 year	94 (6.1)
	≥ 4 year	222 (14.4)
	No response	53 (3.4)
What is the likelihood of your infertility?	Highly unlikely	121 (7.9)
	Unlikely	342 (22.2)
	Possibly	651 (42.2)
	Likely	306 (19.9)
	Highly likely	118 (7.7)
	No response	3 (0.2)
Do you think you should be examined for infertility?	Yes	718 (46.6)
	No	644 (41.8)
Why haven’t you visited a clinic for infertility examination?^b^	There is no time	307 (22.5)
	I am afraid of the gynecologic examination	173 (12.7)
	I feel afraid of discovering the truth	305 (22.4)
	Partner is not cooperative	107 (7.9)
	I don’t know a familiar gynecologic doctor/clinic	304 (22.3)
	If I am infertile, I don’t want another person to know it	89 (6.5)
	Nothing in particular	415 (30.5)
	Other	368 (27.0)

^a^Distribution of responses calculated excluding missing data

^b^This question was asked to women without an experience of a prior infertility examination (n = 1362)

Approximately 338 (21.9%) women believed that women aged > 45 years could have children. Additionally, 47.7% of the participants selected the 40–44 years category as the upper age limit to have children. These results highlight the apparent tendency of most participants to overestimate the possible age of childbearing.

About three-quarters (74.4%) of the women used the ovulation prediction service included in the application. The number of women whose sterile period was < 1 year, 1 year, 2 years, 3 years, and ≥ 4 years was 746 (48.4%), 267 (17.3%), 159 (10.3%), 94 (6.1%), and 222 (14.4%), respectively.

**Fig. 2:**
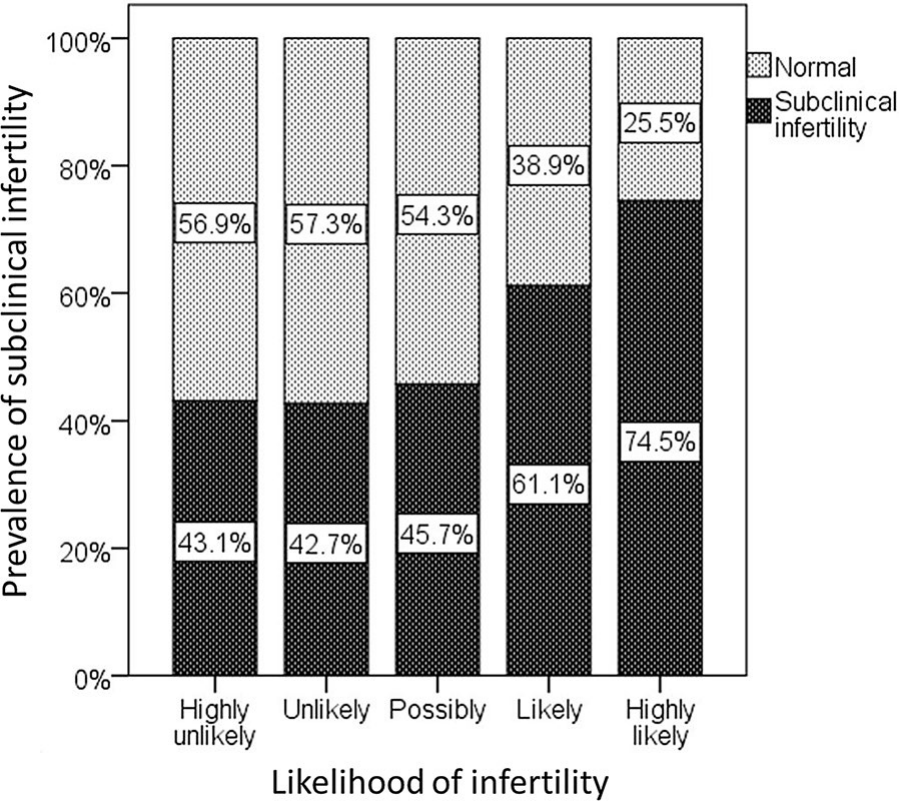
Awareness of infertility and sterile period. *Legend*: Each category of the awareness of infertility corresponds to the results of the question “Do you think you are infertile?” in Table 2. Each bar represents the proportion of potentially sterile women whose sterile periods were more than one year

Regarding infertility awareness, about 70% of women thought the following about the possibility of infertility: (1) highly unlikely (7.9%), (2) unlikely (22.2%), (3) possibly (42.2%), (4) likely (19.9%), and (5) highly likely (7.7%). Among these five groups divided by the likelihood of their infertility, the prevalence of subclinical infertility was calculated as shown in Fig. 2. Among those who gave a negative response to the question on potential infertility (“Never”, “Almost never”), > 40% of women had a sterile period of > 1 year.

Over half of the 1,362 women, who had never been examined for infertility, were aware of the necessity for an infertility examination. The primary reasons for not visiting the clinic were as follows: (i) “no time” (22.5%), (ii) “hesitation to know the truth” (22.4%), and (iii) “an absence of a familiar gynecologist or clinic (22.3%).” Other reasons behind this hesitancy included the following: (1) “the fear of gynecologic examination” (12.7%), (2) “an uncooperative partner” (7.9%), and (3) “the hesitation to let another person know about their infertility (6.5%).” Among the potential answers, the most common subjective reason given for the “other” option was “I do not think myself infertility because of experience of giving birth (N = 80, 5.9%)”, and the second was “financial reason (N = 27, 2.0%)”.

### Relationships between infertility and risk factors

Table 3 summarizes the unadjusted and multivariable-adjusted ORs of infertility for demographic characteristics and fertility experiences. Among the 1,541 women, 742 (48.2%) were infertile with sterile periods of > 1 year. According to the multivariable analysis, multiparity (OR; 1.450, 95% CI; 1.144–1.837, P = 0.002), slightly changed menstrual cycles (variable-length ≥ 7-day difference [OR; 1.536, 95% CI; 1.076–2.193]), and having male partners with smoking habits (OR; 1.383, 95% CI; 1.088–1.759, P = 0.008) were significant risk factors for infertility. With respect to age, three groups, including women aged 35–39 years (OR; 1.885, 95% CI; 1.307–2.719, *P* = 0.001) and 40–44 years (OR; 2.554, 95% CI; 1.490–4.375, *P* = 0.001) and women whose partners were aged 40–49 years (OR; 1.519, 95% CI; 1.030–2.240, *P* = 0.035), had a significantly higher risk of infertility, compared to the youngest group. On the other hand, women’s BMI, smoking history, incidence of abortion or miscarriage, and BMI of male partners were not significant risk factors for infertility.

**Table 3:** The odds ratio of infertility by demographic characteristics and fertility experiences

	Unadjusted odds ratio			Multivariable-adjusted odds ratio^a^		
	OR	95% CI	*p*	OR	95% CI	*p*
*Age(years)*						
16–29	1.0	–	–	–	–	–
30–34	1.222	0.960–1.555	0.104	1.094	0.817–1.466	0.547
35–39	2.371	1.773–3.172	0.000	1.885	1.307–2.719	0.001
40–44	3.360	2.199–5.134	0.000	2.554	1.490–4.375	0.001
45–50	3.944	1.647–9.448	0.002	2.897	1.068–7.859	0.037
*Body mass index (kg/m*^*2*^*)*						
< 18.5	1.0	–	–	–	–	–
18.5–24.9	1.057	0.792–1.410	0.708	0.895	0.650–1.233	0.498
25–29.9	1.236	0.842–1.812	0.279	0.815	0.527–1.260	0.357
≥ 30	2.058	1.193–3.550	0.009	1.364	0.738–2.519	0.322
*Smoking*						
Never or former smoker	1.0	–	–	–	–	–
Current smoker	1.464	1.102–1.943	0.008	1.156	0.832–1.607	0.387
*Delivery status*						
Nullipara	1.0	–	–	–	–	–
Have children	1.782	1.445–2.197	0.000	1.450	1.144–1.837	0.002
*Experience of abortion or miscarriage*						
No	1.0	–	–	–	–	–
Yes	1.767	1.363–2.290	0.000	1.183	0.882–1.585	0.262
*Menstrual cycle*						
Regular	1.0	–	–	–	–	–
Variable length ≥ 7-day difference	1.389	0.991–1.947	0.056	1.536	1.076–2.193	0.018
Interval of amenorrhea of ≥ 60 days	1.374	0.849–2.226	0.196	1.430	0.848–2.409	0.179
*Age of partner (years)*						
18–29	1.0	–	–	–	–	–
30–39	1.667	1.295–2.146	0.000	1.326	0.973–1.806	0.074
40–49	2.671	1.955–3.649	0.000	1.519	1.030–2.240	0.035
50–64	2.529	1.207–5.301	0.014	1.201	0.525–2.747	0.665
*Body mass index of partner (kg/m*^*2*^*)*						
< 18.5	1.0	–	–	–	–	–
18.5–24.9	0.752	0.447–1.264	0.282	0.727	0.408–1.295	0.279
25–29.9	0.876	0.508–1.512	0.635	0.759	0.414–1.392	0.373
≥ 30	1.418	0.715–2.812	0.318	1.187	0.560–2.520	0.655
*Smoking of partner*						
Never or former smoker	1.0	–	–	–	–	–
Current smoker	1.497	1.219–1.840	0.000	1.383	1.088–1.759	0.008

OR, odds ratio; CI, confidence interval

^a^Adjusted for age, body mass index, smoking, delivery status, experience of abortion or miscarriage, menstrual cycle, age of partner, body mass index of partner, and smoking of partner. Multivariate analysis was performed among 1377 women because of 164 missing data

## Discussion

Several previous studies have highlighted the poor knowledge regarding fertility among women of reproductive age [4, 9, 11–15, 17–20, 22, 24, 26–29]. However, in this research field, awareness and attitude regarding infertility among the general population has not been discussed adequately. In this study, we revealed that there were a considerable number of women who continued to try conceiving at home, despite the possibility of subclinical infertility and being aware of it. We also showed that some women hesitated to go to an infertility clinic as they were not familiar with gynecologic examinations.

The number of women who need infertility treatment has been recently increasing in several countries [34, 35]. Among all infertility treatments, ART puts significant economic, mental, and physical burden on the women [36–38]. Furthermore, it has a considerable impact on their daily lives. The increasing number of women with infertility may be attributed to their lifestyle changes, such as delayed parenthood due to higher education, social progress, and late marriage [2–4]. Moreover, several epidemiological investigations from various countries have reported about the poor knowledge regarding infertility among numerous women of reproductive age [4, 9, 11–15, 17–20, 22, 24, 26–29]. This subsequently increases the number of women unexpectedly requiring infertility treatment. Poor fertility awareness is a particularly important concern in some developed countries where declining birth rate is a serious social issue [34, 39, 40].

In this study, the apparent tendency to overestimate the age limitation for childbearing was consistent with those in previously published results [4, 9–22]. Obtaining correct medical information through the internet is accessible for most people in recent times [20, 24]. However, the dissemination of knowledge on fertility and infertility is inadequate among women who need them. Certain background factors, such as the tendency to harbor sensitivity regarding infertility problems and late childbearing by celebrities, also create a significant impact. Hence, imparting sex education might effectively resolve the problem of insufficient knowledge on fertility.

Passet-Wittin, J. identified five categories of determinants of medical help-seeking for infertility: socio-demographic variables, socio-economic factors, reproductive history, attitudes, and psychological factors [41]. Although surveys on psychological factors were limited, anxiety about medical treatment was reported as an important reason for not pursuing medical treatment of infertility [41]. Our results revealed another problem that had not been discussed well in the literature, that is, the hesitancy of visiting an infertility clinic, which was attributed to a lack of familiarity with a gynecologist or clinic and fear of gynecologic examination. Simply put, despite infertility being a characteristic problem in Japan, women faced difficulty in visiting gynecology clinics. The previous surveys on psychological factors were conducted in Europe and US where people have different images of infertility from that in Japan [41–43]. In comparison with people in Europe and the US, Japanese people tend to feel that the topic of infertility is a taboo. Therefore, hesitancy of visiting an infertility clinic may be a unique aspect in Japan. One reason for the aforementioned problem is that women rarely visit gynecology clinics before getting pregnant in Japan. The administration of human papilloma virus vaccines or recognition of women’s health care would enhance their familiarity with a gynecology clinic.

The multivariate analysis in our study indicated “multipara” as a significant risk factor for infertility. Such women may lack sufficient awareness on secondary infertility due to a previous pregnancy. Despite being overlooked for secondary infertility, such women should be aware of infertility in case of a long period of sterility.

Our study has several limitations. First, the participants were selected from among those who used a particular application, thus, raising the possibility of a selection bias. The aforementioned application presented information about pregnancy and was aimed at helping the users conceive. Thus, the participants might have had a stronger desire to bear a child, compared to ordinary women. In addition, there were 10 university students in this study. In general, it is rare for university students to try to get pregnant. However, the number was so small (0.6% of all) that including university students would have little influence on our results. Second, we conducted the study among Japanese women who often harbor negative and sensitive ideas about infertility. This tendency of more women hesitating to go to the clinic for infertility examination or women not wanting to know the truth that they are infertile may have influenced our results. Third, our investigation lacked information about the selected participants’ place of residence. There are several infertility clinics in a city, compared to the few in the countryside. This uneven distribution of infertility clinics might have also influenced the choice to seek infertility treatment. Thus, the differences in this study could not be clarified. Fourth, as there had been no research about medical help-seeking for infertility in Japan, we designed some of the questions about “awareness and attitude regarding infertility” for this study. However, the information might be biased.

## Conclusions

According to our investigation, there may be a significant number of women with subclinical infertility among those trying to get pregnant at home. Furthermore, these women generally hesitate to visit the clinic for several reasons, namely, being unfamiliar with seeing a gynecologic doctor and feeling afraid of discovering the truth. Additionally, our results also indicate that prior experience of childbirth might be a possible risk factor for secondary infertility.

The dissemination of correct knowledge regarding fertility and infertility is insufficient. The presence of a gynecologist who could provide medical advice at an appropriate time might effectively resolve the problem that some subclinical infertility patients encounter, namely; hesitant to go to a clinic because of an unfamiliar gynecologic doctor or clinic. Our study had a small sample size with limited participants. This calls for the need for large-scale studies in various countries.

## Data Availability

The data used and/or analyzed during this study are available from the corresponding author on reasonable request.

## Abbreviations

ARTs: Assisted reproductive technologies
OR: Odds ratios
CI: Confidence interval
BMI: Body mass index
